# SEALDH-II—An Autonomous, Holistically Controlled, First Principles TDLAS Hygrometer for Field and Airborne Applications: Design–Setup–Accuracy/Stability Stress Test

**DOI:** 10.3390/s17010068

**Published:** 2016-12-30

**Authors:** Bernhard Buchholz, Sören Kallweit, Volker Ebert

**Affiliations:** 1 Physikalisch-Technische Bundesanstalt Braunschweig, Braunschweig 38114, Germany; buchholz@csi.tu-darmstadt.de (B.B.); s.kallweit92@web.de (S.K.); 2 Physikalisch Chemisches Institut, Universität Heidelberg, Heidelberg 69120, Germany; 3 Department of Civil and Environmental Engineering, Princeton University, Princeton, NJ 08540, USA; 4 Center of Smart Interfaces, Technische Universität Darmstadt, Darmstadt 64287, Germany

**Keywords:** trace gas measurement, absorption spectroscopy, TDLAS, hygrometer, calibration-free, airborne, field, SEALDH, metrology, traceable

## Abstract

Instrument operation in harsh environments often significantly impacts the trust level of measurement data. While commercial instrument manufacturers clearly define the deployment conditions to achieve trustworthy data in typical standard applications, it is frequently unavoidable in scientific field applications to operate instruments outside these commercial standard application specifications. Scientific instrumentation, however, is employing cutting-edge technology and often highly optimized but also lacks long-term field tests to assess the field vs. laboratory performance. Recently, we developed the Selective Extractive Laser Diode Hygrometer (SEALDH-II), which addresses field and especially airborne applications as well as metrological laboratory validations. SEALDH-II targets reducing deviations between airborne hygrometers (currently up to 20% between the most advanced hygrometers) with a new holistic, internal control and validation concept, which guarantees the transfer of the laboratory performance into a field scenario by capturing more than 80 instrument internal “housekeeping” data to nearly perfectly control SEALDH-II’s health status. SEALDH-II uses a calibration-free, first principles based, direct Tuneable Diode Laser Absorption Spectroscopy (dTDLAS) approach, to cover the entire atmospheric humidity measurement range from about 3 to 40,000 ppmv with a calculated maximum uncertainty of 4.3% ± 3 ppmv. This is achieved not only by innovations in internal instrument monitoring and design, but also by active control algorithms such as a high resolution spectral stabilization. This paper describes the setup, working principles, and instrument stabilization, as well as its precision validation and long-term stress tests in an environmental chamber over an environmental temperature and humidity range of ΔT = 50 K and ΔRH = 80% RH, respectively.

## 1. Introduction

Deploying sensitive instruments using cutting-edge technology outside of a protected laboratory environment often leads to unpredictable changes in the instrument behavior and eventually a lower confidence level of the measurements. Most typical research equipment, including many commercial products, is not designed for particularly harsh conditions often found in the field and in particular for airborne environments. It is not just the environment itself which is different, but also the way field instruments have to be built. Obviously, an optical table with a mass of 500 kg minimizes very efficiently any vibrations, but is not a suitable approach for a field instrument—especially not for an airborne instrument where weight limits are often one of the key restrictions. Other design differences are influenced by the way an instrument is typically deployed in the field, in particular, for remote site operations: here instruments have to be transported, installed and operated with no or very limited opportunities for further maintenance/repairs/optimizations. Similarly, the high operational costs of aircraft campaigns do not allow for time-consuming installation procedures or extensive on-board testing. The entire payload has to be operational within a short installation time. Furthermore, aircraft deployments require a tedious instrument certification to conform to flight certification regulations (depending on the type of aircraft) before each installation, which makes laboratory-like quick component exchanges or on-the-fly modifications very difficult, costly, and often even impossible.

These are just a few examples of why laboratory instrumentation frequently does not meet the expectations under field applications. Even worse is the situation when internal quality control mechanisms don’t report misbehavior of the instrument to the user. Commercial instruments often process internally some maintenance data to guarantee proper working within their specs, but they are usually not accessible for a typical user lacking a deeper knowledge needed to understand or even assess effects hidden in the behavior of a black box type instrument. Contrarily, scientific research instrument developers, having in theory full access to all data, frequently avoid investing a large amount of development time in internal quality control mechanisms. From a scientific point of view, however, this situation of not being able to access or not having high quality control mechanisms linked to the measurements is not acceptable as the scientific findings often rely on accurate data with a defined measurement uncertainty and a sufficiently solid understanding of the instrument behavior. This holds particularly for water vapor measurements [1,2,3]. Airborne/field water vapor measurements have been challenging hygrometers for a long time. Despite the tremendous development efforts, deviations between the instruments remain significant. This was e.g., shown in 2007 when an international comparison exercise, “AquaVIT” [4,5], was organized to compare the world’s best airborne hygrometers. Even under well-controlled, quasi-static measurement conditions, the instruments deviated by up to ±10% (relative) from the group mean. Since the mean value was not metrologically traced-back (due to the lack of an reference instrument which can serve under these conditions as a metrological transfer standard), this signaled to the atmospheric communities that two measurements from two different hygrometers could be off by 20% which is for many atmospheric questions not acceptable and called for improved hygrometers. Other, less representative studies such as [6,7] report similar results.

In this paper we present a new, autonomous, and highly robust direct tuneable diode laser absorption spectroscopy (dTDLAS)-based, first principles, and thus calibration-free hygrometer, which has a very extensive internal quality and instrumental status control system (ISCS). We will discuss the basic spectroscopic working principles, as well as our new holistic ISCS which encompasses more than 80 internal instrument parameters, so called “housekeeping data”. ISCS allows close monitoring of the instrument behavior particularly under varying experimental conditions and thus enables a much higher trust level for the data. In particular, we discuss in closer detail the active spectral stabilization module which is highly responsible for the good performance data, as well as possible improvements that can be achieved with it. Finally, we discuss SEALDH-II’s precision and accuracy statements in harsh environments derived from a long-term validation in an environmental simulation chamber by varying ambient air temperature and humidity where all active compensation, correction, and preventive instrument control measures are challenged to compensate these external impacts.

## 2. SEALDH-II Overall Description

**Figure 1:**
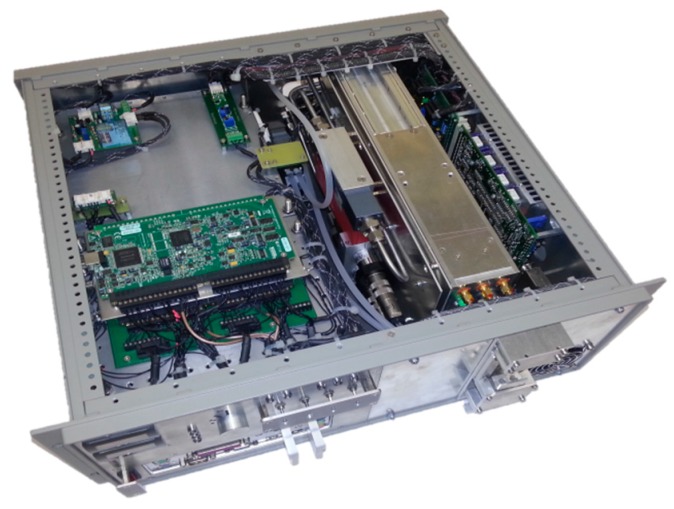
Photo of the SEALDH-II instrument (top cover removed). Several electronic boards are visible, as well as the large metal “opto-electronic laser box” on the right side (for further details see text).

Figure 1 shows a top view photograph of the SEALDH-II instrument. The top cover of the 19” 4 U instrument frame is removed. The front panel comprises several displays and switches for convenient communication with a user. The digital remote control interface (e.g., for boot signals from aircraft) as well as the gas inlets are on the backside panel. Inside on the left side is a three-layer stack of horizontally oriented trays for electronics. The lowest level contains power supplies and an Intel Atom based computer. The second layer contains two SSD hard drive, several transformer electronics as well as the data acquisition (DAQ) card for all spectroscopic signals (NI USB-6361 X Series, National Instruments, Austin, TX, USA). The top layer (which is visible in the pictures) contains additional DAQ cards (two NI USB-6210) and supplementary electronics (LCD display drivers, aircraft interface module, etc.). The green small circuit board on the right corner of the stack contains humidity (SHT2x), pressure (mpxa6115a), and temperature (SHT2x) sensors, which will play an important role in the following sections (e.g., in Figure 10 as the sensor for the ‘19” slide-in temperature’ and ‘internal relative humidity’). Next to the three-layer stack is a gas handling system, which contains a heat exchanger, two pressure sensors (one before the heat exchanger and one at outlet of measurement cell to measure the pressure drop), a compact flow controller (0–10 SLPM), and specially designed temperature converter electronics (differential + absolute measurement of the temperature gradient along the heat exchanger). The entire piping before the measurement cell is made of electro-polished stainless steel. This effectively minimizes hysteresis effects caused by absorption and desorption of water onto and off the surfaces. The heat exchanger assembly thermalizes the provided gas flow to the internal instrument temperature. Pre-thermalization and conditioning of the gas before the optical measurement cell serves to guarantee a well-defined and homogeneous gas temperature in the internal cell. This is very important for an accurate spectroscopic evaluation of the water signal with low uncertainties, and is just one of many “preventive measures” to precisely control or log the entire measurement conditions within SEALDH-II.

The stainless-steel colored metal box is the “opto-electronic laser module” (OELM), which contains e.g., three transimpedance amplifiers for photodiodes, converter electronics, heating elements, several temperature sensors (e.g., for frame-, cell-, and gas temperature), fiber-splitters and light attenuators. Furthermore, the OELM is equipped with a new purge-able and entirely supervised (temperature, pressure, humidity, supply voltage, heat conductivity, etc.) laser mount, which serves to minimize parasitic absorption offsets by residual water vapor outside the measurement cell (detailed description in [8]).

Directly attached to the chassis on the right side is a laser driver (LD) module (ITC-102, Thorlabs, Newton, NJ, USA) with some additional electronics (LD interface, DAQ-to-LD voltage matching, analog pre-filtering for control signals, temperature converter for LD temperature measurement, two fans for LD). The laser driver module uses the instrument chassis as a heat sink to increase the total thermal mass, which slows down a possible temperature drift and will be an important issue during the ambient temperature impact validations in this paper. To allow an aircraft certification of SEALDH-II, any flammable plastics had to be avoided, which was one of our design drivers.

In the following, we describe the core spectrometer and the most important supplementary modules of SEALDH-II. In the interest of a compact paper, we preferred a compact description of all developed techniques and modules. The more detailed descriptions of modules for certain complicated techniques like auto instrument self-start or autonomous instrument self-characterization are referred to specialized, subsequent papers.

### 2.1. SEALDH-II Core Spectrometer

The core spectrometer function of SEALDH-II is based on direct Tunable Diode Laser Absorption Spectroscopy (dTDLAS), which was recently described in more detail elsewhere [9,10,11], especially for airborne hygrometry [4,12,13,14,15], just to refer a few important publications. We use a proprietary, and not yet common, first principle evaluation method [16,17,18,19], allowing a direct retrieval of the final concentration without an initial or repetitive calibration procedure of the spectrometer response function using a humidity standard. A first version “SEALDH-0”, not suitable for airborne and autonomous operation, was developed and used for stationary laboratory studies and described elsewhere [20]. The physical principles of the “core spectrometer” are already described in detail in this previous paper [20], so we describe the core functions in the present paper only very briefly where it is indispensable for understanding the concept of SEALDH‑II.

#### 2.1.1. Non-Calibrated Direct TDLAS (dTDLAS)

**Figure 2:**
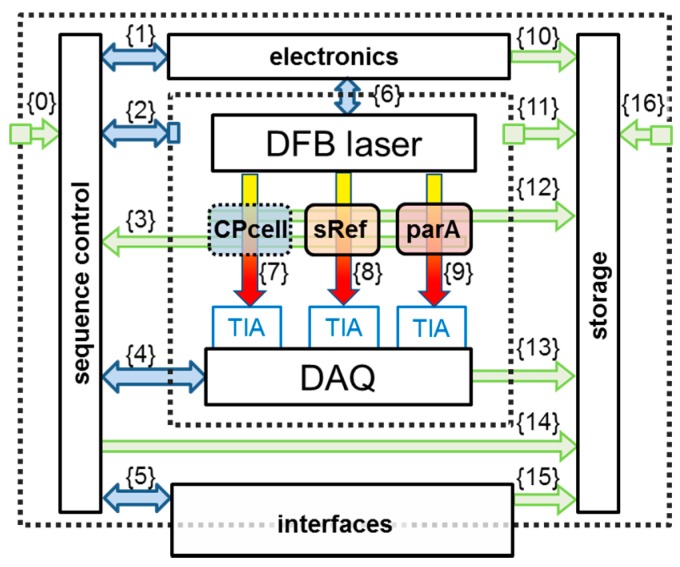
Graphical representation of the data flows inside the SEALDH-II instrument. The actual spectroscopic measurement path in the closed-path cell “CPcell” is embedded in a network of supplementary data flows for various instrument control purposes (marked with {numbers}). Details are described in the text.

Figure 2 shows a functional sketch of the spectrometer’s building blocks. These are: a distributed feedback (DFB) diode laser, the closed-path optical absorption cell (CPcell), three transimpedance amplifiers (TIA), a DAQ card, and further peripheral electronics. The functional principle of TDLAS relies on the determination of (a) the initial light intensity *I*_0_(*λ*), before the absorption path (typically being in the few mW-range) and the transmitted light intensity *I*(*λ*) after the absorption region. The relationship between these two entities can be described by the *extended* Lambert-Beer equation (Equation (1)), Figure 2 is just a duplicate of Figure 1.

(1) $$I\left(\lambda \right)=E\left(t\right)+I_{0}\left(\lambda \right)\cdot Tr\left(t\right)\cdot exp\left[-S\left(T\right)\cdot g\left(\lambda -\lambda_{0}\right)\cdot N\cdot L\right]$$

which includes possible disturbances of the standard Lambert Beer equation by stray light-type background radiation *E*(*t*) or additional broadband transmission losses *Tr*(*t*), e.g., by contaminations on optical windows. The exponential term embraces the line strength *S*(*T*) of the selected molecular transition, the line shape function $g\left(\lambda -\lambda_{0}\right)$, the absorber number density *N* and the optical path length *L*.

The H_2_O volume mixing ratio *c* (metrologically correct to be correctly designated as *amount fraction*) can be retrieved from the integrated line area of the absorption profile of a single rotational–vibrational H_2_O transition by combining Equation (1) with the ideal gas law. This leads to:

(2) $$c=-\frac{k_{B}\cdot T}{S\left(T\right)\cdot L\cdot p}\int \ln\left(\frac{I\left(\nu \right)-E\left(t\right)}{I_{0}\left(\nu \right)\cdot Tr\left(t\right)}\right)\frac{d\nu }{dt}dt$$

The individual contributions to Equation (2) are: constant entities like the Boltzmann constant $k_{B}$; the optical path length *L*; molecular constants like the line strength *S*(*T*) of the selected molecular transition; the dynamic laser tuning coefficient $\frac{d\nu }{dt}$, which is a constant laser property; continuously measured entities, i.e., the gas pressure (*p*), the gas temperature (*T*) and the line area derived from the ratio of the photo detector signals for the initial *I*_0_(*λ*) and the transmitted light intensity *I*(*λ*). The initial intensity signal *I*_0_(*λ*) is retrieved from the measured transmitted light intensity signal *I*(*λ*) via an absorption model. The determination of the dynamic tuning coefficient is based on an Airy signal generated by passing the laser light through a planar, air-spaced etalon with a well-known thickness [16,21]. In other words, we directly link the laser tuning coefficient to the SI unit “length”. An application dependent optimized selection of an absorption line is a critical step in the instrument design process and is described e.g., in [20,22] or [23]. Molecular line parameters can be extracted from large molecular parameter databases as described in [24]. Line parameters of the transition used in SEALDH-II (energy levels: 110–211, ro–vibrational combination band near 1370 nm) are used in numerous previous applications [4,11,15,25,26,27,28,29,30,31,32], and have thus been trustfully determined by independent measurements, e.g., [33].

#### 2.1.2. Data Processing and Evaluation

**Figure 3:**
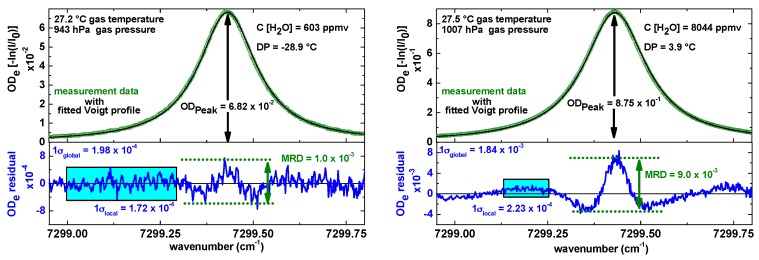
Typical pre-processed absorption signals from SEALDH-II for two different concentrations after baseline, offset, and transmission correction. The green dots are the measurement data, the black line is the multi-line Voigt fit, and the blue line is the associated fit residual. The laser modulation frequency was 140 Hz; 20 individual raw scans are pre-averaged yielding 7 [H_2_O] measurements per second. Abbreviations: DP = dew point of H_2_O, C = concentration of H_2_O, MRD = maximum residual deviation (see definition in [20]), OD = absorbance.

Figure 3 shows two raw absorption signals (one at 600 ppmv and one at 8000 ppmv H_2_O in air) from the SEALDH-II measurement cell after baseline, offset, and transmission correction. The measurement conditions are in both cases close to room temperature and standard pressure. This means that the absorption lines for typical flight operation will be narrower due to reduced pressure broadening at the reduced pressures of higher altitudes. These raw absorption scans (green dots) were recorded with 140 Hz scan repetition rate and pre-averaged by co-adding 20 subsequent spectral scans which yielded an effective spectrometer repetition rate of seven measurements per second (7 Hz). Depending on the pressure and therefore on the broadening of the absorption line, just 20%–40% of a laser cycle contains the actual H_2_O relevant measurement data. The rest is used for the retrieval of *I*(*λ*), the so called baseline, and for background stray light detection. This leads to a total integration time of 2.2 ms (30%) at 140 Hz and 44 ms at 7 Hz. In other words, the actual time resolution of SEALDH-II is purely defined by the gas exchange rate discussed in Section 2.1.3.

The line area needed for Equation (2) is derived by fitting a set of Voigt line shapes to the measured signal. This multi-line set is necessary since the absorption line of the “target” transition (energy levels: 110–211, ro–vibrational combination band near 1370 nm) is surrounded by 18 weaker transitions absorption lines which have to be taken into account in the physical model for first principle evaluations. In order to maximize the fit stability, we consequently minimize the degrees of freedom of each absorption line peak by pre-computing as many line shape parameters as possible, i.e., collisional broadening from the air-broadening coefficient and the measured total pressure, Doppler broadening from the measured gas temperature, etc. The result of such an optimized fit strategy is depicted in Figure 3: the black lines in each graph in Figure 3 indicate the fitted multi-line Voigt profiles. The blue lines, depicted below, are the residuals between the measured data and the spectral model function. The visible deviation in the residual for higher concentrations was previously discussed in [20], but can be explained by line shape deficits of the Voigt profile function used for this evaluation. The Voigt profile is not capable of precisely describing all higher order spectroscopic effects such as speed-dependence [34] or Dicke narrowing [35], which become significant at the high signal-to-noise ratios (SNR) of our dTDLAS spectrometer. This small “disadvantage”, which leads to a maximum relative systematic error of about 4% at 50 mbar and less than 1% at atmospheric pressure, is deliberately taken into account in order to gain the advantage of a rapid and predictive fit performance: in the standard operation mode of SEALDH-II we avoided higher-order line shapes beyond Voigt, in order to secure the Voigt shape benefit to pre-calculate all line parameter (temperature-, self-, and foreign broadening and the spectral line position) which leads in the line fitting process to a reduction (see [20]) to just one single degree of freedom: the line area. This yields—even under harsh conditions—a much more stable, reliable and rapid fitting process with a better defined uncertainty for the entire atmospheric H_2_O range (3–40,000 ppmv, 100–1000 hPa) and is preferable for our main application targets of SEALDH-II, e.g., airborne field measurements. For higher order line shape profiles, highly accurate molecular transition parameters are missing up to now which would hinder accurate pre-calculation of most of the fitting parameters. On the other hand, since SEALDH-II stores all raw scans to SSD, it is always possible at a later stage to reanalyze the data (e.g., after the flight) as soon as an improved line shape model and a complete set of molecular parameters become available.

#### 2.1.3. Extractive, Multi-Pass Absorption Cell

The extractive, closed-path cell (CPcell Figure 2) is a successor of the early stage development cell, published in [36], which was used in the previous mentioned laboratory study for SEALDH-0 [20]. The new cell has a compact, stiff, miniature White type [37] design that folds the absorption path (total length approx. 1.5 m) with three mirrors at approx. 7.5 cm base distance. The cell has an innovative fiber feedthrough [38] which guides the light into the cell via a lens-less direct fiber coupling. The fiber feedthrough reaches a very low leakage rate of 1.9 × 10^−6^ hPa·L/s and avoids contamination offsets due to ambient air humidity. This direct fiber coupling efficiently avoids fringing and misalignments since there are no additional optic elements such as lenses needed. White cells with this design are also quite robust against mirror degradation or slight misalignments, which is fairly important for airborne deployment. The airflow into the cell is pre-heated/-cooled before it reaches the cell by a heat exchanger to avoid temperature inhomogeneity which would cause deviation according to Equation (2). With this approach we minimize temperature fluctuations below 0.1 K even during aircraft operation. The temperature within the cell is simultaneously measured by an accurate (ΔT = 0.3 K) PT100 sensor (form factor: SMD 0805: 1.4 × 2.2 × 0.6 mm^3^) for rather slow temperature changes (t_50%_ approx. 2.5 s) and with a thin (0.5 mm diameter) E-type thermocouple for faster temperature changes (t_50%_ approx. 0.5 s). The gas flow is measured and regulated by a special low pressure drop mass flow controller which is attached after the cell. By using the cell volume of 300 ccm, a bulk flow approximation, typical conditions of 200 hPa outside pressure, and 7 SLPM as flow through the SEALDH-II sensor, we estimate the cell flushing time to be around (0.3/(7 × 1013/200) × 60 s) = 0.5 s. It has to be kept in mind, that this “bulk flow model” is not directly applicable to turbulent flows; parts of the cell will be flushed faster, parts slower. Hence, this value has to be seen as an estimate.

#### 2.1.4. “Non-Calibrated” vs. Calibrated Instrumentation

The results of the international inter-comparison for airborne hygrometers (AquaVIT [5]) in 2008 showed, briefly summarized, that the different instruments’ absolute response deviations span ±10% around the group average even for the subgroup of the so called core instruments, which contained some of the world’s best performing airborne hygrometers with extensive multi-year validation experience.

The maximum deviation within this subgroup could yield up to 20% in the atmospherically relevant H_2_O concentration (1 to 200 ppmv) range. This leads to the question if it is really related to a poor long-term stability of those instruments rather than to the absolute accuracy and long-term stability of their various sources used for instrument calibration. Solving this problem requires an extensive absolute validation of instrument as well as calibration source accuracy and stability. On the other hand, an absolute, first principles measurement technique, e.g., like our calibration-free dTDLAS approach, could—after a careful absolute validation—eliminate these problems associated with tedious and frequent instrument calibration. A calibration-free instrument requires an excellent long-term stability, since long-term-instabilities together with the instrument resolution and noise would limit the achievable accuracy of the instrument. On the other hand, a calibration-free technique still allows the measurement for validation and even calibration. If the performance of a calibration reference is much better than a calibration-free instrument, it is always possible to enhance the instrument performance by calibrating it with a reference standard such as [39] at any time even after a campaign as demonstrated in [26].

This also spurs interest in an approach which needs as little calibration effort as possible and raises the question about the accuracy level which can be achieved without any calibration at all. Special versions of dTDLAS offer this possibility, which can be deduced from a closer look at Equation (2) which fully describes the evaluation principle used in the SEALDH-II instrument. Equation (2) does not contain a single calibration parameter which would be used to “adjust” the instrument to the values given in a calibration process, e.g., to modify the instrument’s linearity or offset parameters. This indicates that Equation (2) paves the path to an accurate, absolute response instrument if all parameters, shown in Equation (2), are determined with a sufficient accuracy. The final absolute water vapor values in SEALDH-II’s measurement cell are retrieved only by using this equation and measurement data according to the equation itself. A calibration-free approach is particularly valuable for gas components which are not available as stable gas mixtures in bottles or which are difficult to be dynamically generated with respect to small cost, size, and/or power requirements. This is particularly important for strongly adsorbing species like water vapor or ammonia, or for highly reactive species like HCl, HF, NO_2_, etc. In these cases, the calibration process is extremely difficult and therefore inaccurate, e.g., due to ad/absorptions effects on nearly all surfaces. Calibration procedures with (dynamic) reference generators are often available for and compatible with lab scenarios, but then these generators tend to be quite large, costly and the calibration itself time consuming, due to the long waiting time for the adsorption/desorption equilibria. Therefore, this laboratory calibration approach is frequently incompatible with field science situations, where neither time and space, nor excellent control over the calibration boundary condition is available. Eventually, this leads to large, often very difficult to quantify uncertainty contributions. In such scenarios, calibration-free optical techniques like dTDLAS offer a clear advantage in the necessary effort but also in the absolute performance and trust level, as otherwise the instrument calibration process has to be performed in a fraction of the typical laboratory calibration time and with a much smaller and thus less accurate mobile reference generator.

#### 2.1.5. Brief Consideration of the Instrument Uncertainty

The physical model of dTDLAS (Equation (2)) facilitates the direct calculation of an absolute uncertainty budget containing individual contributions of all parameters in Equation (2). For the case of SEALDH-II, this leads to the following contributions:

The temperature sensors (PT100 and thermocouple type E) are calibrated at Physikalisch Technische Bundesanstalt (PTB, the German National Metrology Institute) against a metrological transfer standard, a reference platinum resistance thermometer type 162CE (PRT-25, Rosemount, Shakopee, MN, USA) with an uncertainty of 1.5 mK. Nevertheless, the accuracy of the gas temperature measurement is not limited by the electronics and converters but rather by the well-known fact that temperature measurements in moving gases suffer from many additional issues. Thus, we assume a quite conservative gas temperature uncertainty of 1 K (0.3%). Similarly, the gas pressure sensor has a resolution of 0.02 hPa and a long-term drift of less than 0.5 hPa per year. Therefore, we use 1 hPa as conservative uncertainty estimate. The optical path length in the closed-path cell (uncertainty 15 mm (1.1%)) was primarily determined via a Zemax based ray tracing simulation and a mechanical mirror distance measurement as well as validated by using a CH_4_ reference gas with a known concentration. The uncertainty for the used H_2_O absorption line strength is 3.5% [33]. The last uncertainty contribution is the fit process and laser tuning uncertainty. A general statement in a metrological sense is not simple since the uncertainty depends on local effects in the spectra, pressure range, concentration level, and the number of absorption lines fitted. Thus, we estimate, based on our experience from comparable dTDLAS setups, the uncertainty related to the fitting process in total (tuning + fit process) to be in the range of below 1.5%. As a conservative approximation for SEALDH-II’s evaluations, we use 2% for the fit and 1% for the tuning. This leads to a total uncertainty of 4.3%. The largest relative contributors can be ranked as follows: The total uncertainty is dominated by the line strength uncertainty (66%), followed by the fitting uncertainty (21%), as well as the optical path length uncertainty (7%). The offset uncertainty is defined in a calibration-free dTDLAS system like SEALDH-II by the capability of minimizing and determining parasitic effects, see [8]. SEALDH-II has a relatively low offset uncertainty contribution (approx. 10^−5^ to 10^−6^ relative to the maximum spectroscopically achievable concentration) due to the novel parasitic water vapor treatment, which is described in detail in [8]. This paper describes an integrated concept how to setup (hardware and software) a diode laser system to minimize, determine and correct for parasitic water vapor effects down to the 10^−4^ absorbance (OD) level in a remote or airborne application. In addition, it points out the limits of the typical “purging with dry nitrogen” approach which is commonly used in laboratories. The total uncertainty of SEALDH-II is described by a term proportional to the absolute mixture fraction (4.3%) and by an absolute offset uncertainty of ±3 ppmv.

To achieve such an uncertainty even in a calibration-free dTDLAS approach requires a large amount of active controlling modules inside the laser spectrometer to precisely stabilize, analyze, or compensate the measurement conditions. Furthermore, a variety of independent monitoring parameters is advantageous to facilitate the assignments of the instrument behavior and to achieve a measurement uncertainty closer to the one in a metrological sense [40]—especially under the harsh condition in field deployments. Several of these additional control mechanisms are described in the following sections.

### 2.2. Supplementary Data Acquisition and Handling

Figure 2 shows the entire spectrometer as functional units with their data/information streams {i}; blue streams are active steering and control data, green streams are measured data. On the right side of the discussed closed-path cell (CPcell) {7}, there are two additional spectroscopic channels. One is used for the spectral stabilization (sRef) {8} of the spectrometer, which will be explained later, the other (parA) {9} to determine and compensate parasitic absorption effects [8]. The electronic module contains all diode laser relevant spectrometer electronics such as frequency generator, laser driver, diode laser temperature controller, etc. to control the diode laser via {6}. The DAQ module captures all data from the spectroscopic signal paths and contains photodiodes as well as associated transimpedance amplifiers, the Digital to Analog (A/D) and Analog to Digital (D/A) converter/digitizer cards and power-supply filters to reduce electrical noise. The sequence-control scheme interacts with all modules via bus connections designated as {1}, {2}, {4}, {5} in Figure 2.

The interface module processes all external data from outside of the instrument, such as communication via Local Area Network (LAN), the aircraft related communication, as well as from the user via a front panel. The storage module saves, whenever possible, raw data of all supplementary measurements including timing and channel information. Raw data are also stored from all spectroscopic channels (photodiodes) in order to allow a full post analysis with improved spectroscopic line parameters or with new line shape profiles. In total more than 80 independent parameters are collected and saved to SSD drives, which allows depicting and documenting of the entire instrument status at relatively high time resolution at any time during a measurement deployment.

Data streams {0} and {16} contain information about the environmental conditions such as ambient pressure, temperature, power supply status, etc. The inner dashed frame represents the encapsulated opto-electronic laser box (see picture in Figure 1), which is by its design shielded from the electromagnetic-radiation of other parts. This box is controlled by {2} and data such as internal structural and gas temperatures, pressures, humidity, etc. are recorded via {11}. All important decisions done by the sequence control are stored for post analysis {14}, as well as the status/set points etc. of the interface {15} and electronics {10}. The streams {3} and {12} contain beside the typical supplementary spectroscopic measurements (gas pressure and gas temperature) data such as temperature of the (sRef) detector as well as additional parameters like the flow rate through the gas handling system. In summary, all the data which could help assessing malfunctions, deviations or unusual status of SEALDH-II during operation are captured to generate a complete and detailed overview of the instrument behavior, which is very helpful to evaluate the performance in a laboratory scenario, but which is particularly important in the harsh and highly variable boundary conditions in field situations or in flight.

### 2.3. Instrument Scenario Recognition, Handling, and Preventive Measures

**Figure 4:**
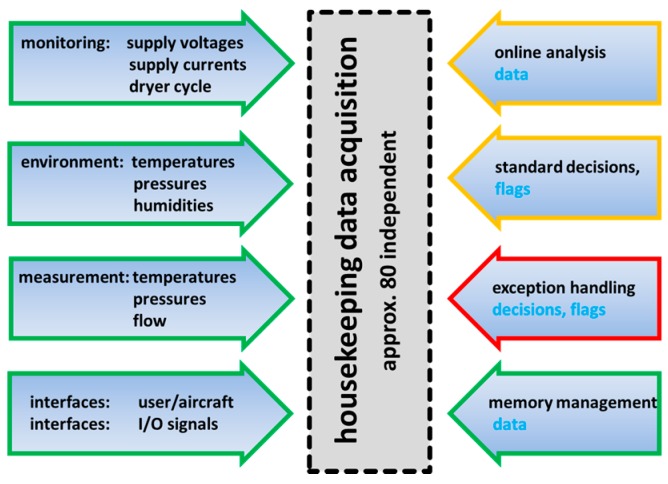
Classification of the values acquired by the control sequence software: Data from sensors and interfaces, output of the built-in algorithms, exception handling, and memory management.

The primary goal of SEALDH-II is to deliver reliable measurements under all circumstances. Reliable in this sense means within well-defined measurement uncertainties and with a distinct statement that the data are not affected by any instrument malfunction. To ensure that, SEALDH-II collects a large amount of 80 independent “instrument health” or “housekeeping” parameters which are classified in Figure 4. These parameters are grouped in different categories such as “monitoring functions”, “environmental parameters”, “spectroscopic measurements”, and “interfaces”. By gathering all of this information, SEALDH-II allows a highly resolved categorization of every individual data point in status classes like “caused by an external impact” or “is a real measured effect”. Besides this monitoring function, SEALDH‑II analyses data to actively control the instrument, e.g., to perform an active, high-resolution spectral stabilization of the diode laser, which will be explained in detail later. All decisions, made by the instrument control software as well as all handled exceptions, are recorded to trace back each malfunction and all control activities. The remaining category in Figure 4 is the memory management which contains functions like a smart, situation dependent dTDLAS raw signal pre-averaging algorithm to slightly compress (binary) the data stream in order to allow for longer instrument operation (before the SSD drives fill up with the large amounts of raw data). The memory management also provides an information reduction and compression tool for cases where the remote network connection to the instrument is slow (e.g., GPRS). By combining all collected data and functionalities, SEALDH-II can handle a large variety of difficult situations occurring in a field or flight scenario. Table 1 lists such typical scenarios with associated capabilities; a character in parenthesis implies that a general statement cannot be given and therefore depends on the situation.

To illustrate Table 1, we will pick one scenario in a brief case study: We assume an external temperature variation as an ambient disturbance (e.g., exposed to sunlight (field), cabin temperature change (aircraft) etc.): A temperature variation has an impact on accuracy (thus “a” in table row) and precision (thus “p” in table row). It could cause a system crash (“c”) or even cause permanent damages (“d”) if there is internal condensation in the measurement cell or instrument. Several temperatures in SEALDH‑II monitor and document (“m”) this situation. Impacts are actively corrected (“cor”) as we will discuss later on with the example of the spectral stabilization. In addition, preventive (“p”) measures are integrated in the instrument such as an optimization of the thermal masses to minimize the consequences of a temperature variation (will also be explained later on in more detail). Table 1 summarizes the most important scenarios as well as SEALDH-II’s associated handling capabilities though not all of them will be discussed in this paper in full detail, due to space restrictions.

## 3. Active spectral Stabilization of the Instrument

### 3.1. Principle Design and Setup

The wavelength of a DFB diode laser can be tuned by laser current and temperature modulation. Current tuning occurs rather fast by ohmic heating within the active lasing region of the semiconductor chip and typically yields a wavelength tuning of approx. 2 cm^−1^ per 100 mA for modulation frequencies up to 1000 Hz. Variation of the entire semiconductor temperature is rather slow due to the significant thermal mass of the diode laser assembly and the Peltier element (Thermo-Electric Cooler, TEC) typically used for controlling the laser temperature. The limited cooling and heating power of a Peltier element and the limited heat conductivity between laser chip and TEC lead to typical temperature adjustment times around 1 to 10 s (sometimes even longer). The achievable temperature tuning range for a DFB laser is approximately 0.5 cm^−1^ per Kelvin with an accessible temperature span of 20 to 50 K with simple TECs.

In most diode laser spectrometers, the laser temperature is stabilized via a Proportional, Integral, Differential (PID) controller to keep the so called “center emission wavelength” at a certain value. Simultaneous current modulation thus allows a small but fast wavelength modulation around this center wavelength. This spectral modulation range around a center wavelength will hereafter be called “scan”. As shown in the spectrometer schematics (Figure 2) the modulated light is transmitted though the measurement area {7} and two supplementary channels {8}, {9} and then guided to three independent detectors. The photocurrents from these detectors are converted into voltage signals via transimpedance amplifiers (TIA) and then acquired by an analog-to-digital converter (ADC) data acquisition (DAQ) card. One recorded wavelength scan comprises typically 2000 individual, time-equidistant data points, which are measured with 16‑bit digital resolution. The “natural unit” of a scan is therefore photocurrent vs. time, which is translated by the acquisition electronics into to “voltage vs. pixel”. The time difference between two pixels can be used together with the dynamic tuning of the used diode laser to calculate the wavelength difference between two pixels. In other words, a wavelength scan represents a certain recorded spectral range, which is stable as long as the temperature of the laser chip doesn’t change. SEALDH-II records 140 of these individual wavelength scans per second for each of the three spectroscopic channels (Figure 2).

**Figure 5:**
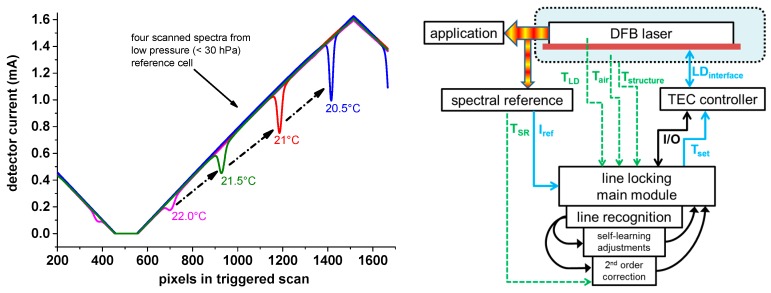
**Left**: Consequence of a diode laser chip temperature variation, demonstrated at for different, independent spectra which are combined in this figure. These spectra are acquired after the laser light transmitted a low pressure reference cell filled with a mixture of H_2_O and air (exact knowledge of the water vapor mixing ratio is not needed). The temperature variation causes a shift in the scanned wavelength range which eventually leads to a shifted line position in the spectra. Due to the nonlinear laser tuning over the spectral scan a position shift—if not corrected for—leads to a systematic H_2_O response variation of the instrument; **Right**: Sketch of the important modules and data flows needed for the spectral stabilization.

Figure 5 (Left) visualizes what happens if the laser chip temperature is unstable and drifts: a laser chip temperature change shifts the absolute spectral position of the wavelength scan range which results in an apparent shift of the absorption line within the scan. A typical spectral scan looks like the red marked absorption line, where the minimum of the absorption line is at pixel position 1200, which corresponds to a laser chip temperature of 21 °C. The more the absorption line is at the lower end (pixel 550), the higher the noise, and the more non-linear is the local tuning coefficient of the laser. Shifting the absorption line close to the maximum of the scan (=pixel 1500) should also be avoided since the Lorentzian wings of the absorption line, which carry valuable spectral information, are then pinched off. This information is needed to retrieve the spectral baseline (*I*_0_(*λ*)) from this scan, which is the signal if no absorption happened.

As mentioned, most spectrometers use a temperature controller to keep the temperature fixed which usually works with high quality laser drivers in protected laboratory environments. However, this changes if the laser driver itself is exposed to temperature changes. Generally, all electronic devices need an absolute reference (voltage) to stabilize another absolute (electronic) signal. This absolute reference is typically realized via a so-called Zener diode which has a well-defined and stable reverse voltage. However, like in all semiconductor PN transition regions, this reverse voltage itself also depends on temperature, so quite advanced measures are needed to compensate for that. At a second order level, every electronic part has small temperature dependencies. Together, these are the main reasons why external temperature changes act on the electronics components of each setup and in our case cause temperature dependent drifts in the spectral wavelength scans. These drifts lead to changes in the H_2_O line position within the scanned range and lead to significant changes in the line shape, caused by the non-linear dynamic wavelength tuning of the laser, which are very difficult to compensate for completely. The absolute accuracy requirements for laser diode temperature controllers are therefore quite high, since an absolute temperature change of only 0.1 K already causes a shift of the spectral scan of 0.1 cm^−1^, which is on the order of 5% of the total scan width and 200% of the line width (HWHM = half width at half maximum) at 500 mbar. A high resolution spectral stabilization is thus of highest importance for the stability and accuracy of the instrument. This is especially important if an instrument like SEALDH-II is deployed in the field or in an aircraft where large ambient temperature changes can lead to center wavelength shifts in the order of 0.5 cm^−1^ which prevents an evaluation with defined uncertainties.

### 3.2. Spectral Spectrometer Stabilization Algorithm

A solution for minimizing this drift is a precise absolute spectral stabilization of the diode laser. This can be realized by adding another spectroscopic channel with a low pressure reference cell (see {8} in Figure 2), photodiode, electronics and software to the spectrometer. With this additional channel, the spectral drift of the laser can be linked to an absolute spectral position of a very narrow H_2_O reference line in the low pressure gas cell. Figure 5 (Right) shows the essential parts of the line locking module which will be described in the following section.

The diode laser is connected to a TEC controller which tries to keep the temperature of the diode laser (T_LD_) as stable as possible—similar to a standard TDLAS spectrometer. The light of the diode laser is divided into two paths, one for the target application (e.g., measurement cell) and one for the spectral line-locking reference cell, which, within SEALDH-II, is a low pressure (approx. 30 hPa), fiber coupled, single path (approx. 4 cm), absorption cell. The absorption signal of the reference cell looks after digitization like one of the scans in Figure 5 (Left) and is further processed by the line locking software module. The absorption line of the low pressure cell is quite narrow and of almost constant line width, so that the absorption peak (=local minimum) is relatively easy to find by a minimum search algorithm. However, just using the position of the local minimum would mean that the entire line locking depends on a few pixels, acquired with a fast (2 MS/s) A/D converter. Therefore, it is more effective to use a-priori information about the absorption lines which are similar in all scans. Practically, the line recognition algorithm retrieves the base line (=signal if no absorption happened) first, which is straightforward since the line is quite narrow compared to the entire scan (ratio approx. 1:15). Subsequently, the area of the absorption line is detected and the first derivative calculated. A linear regression in the vicinity of the detected absorption line position is calculated; the intersection with *y* = 0 is the desired position of the desired minimum. The benefit of this approach is that approximately 50 pixels are used to calculate the line position, which efficiently averages out noise. The position of a spectral line at a given pressure is a molecular constant and therefore absolute and stable over time. For very high accuracy line-locking, the pressure induced line shift has to be taken into account, as the pressure in the low pressure reference cell is not completely stable due to the temperature change of the low pressure reference cell itself. For example, a temperature increase of the reference cell leads to a pressure increase of the reference cell (ideal gas law). One solution would be to thermally stabilize the reference cell which needs additional electronics, power, and causes costs. SEALDH-II, however, uses a different approach, performed in the “2nd order correction” module (Figure 5). The pressure change in the reference cell can be calculated with the ideal gas law and the knowledge of the reference cell temperature. It is on the order of 0.1 hPa/K at room temperature and at 30 hPa gas pressure. Pressure shift coefficients of absorption lines are also a molecular constant, which can be looked up in a database like HITRAN [24]. The pressure shift coefficient of the used absorption line (−8.4 × 10^−6^ cm^−1^/hPa) has an uncertainty of 10%, which would lead with a temperature uncertainty of the reference cell of 1 K to an absolute line position uncertainty in the correction of 1.2 × 10^−6^ cm^−1^. This corrected position information is retrieved 10 times per second and used in a feedback loop to change the set temperature (T_set_) of the TEC controller (PID), which is in charge of the laser chip temperature.

However, this doesn’t work directly since the entire feedback loop (from line position detection, TEC element temperature change, laser chip temperature change to actual detected laser wavelength shift) shows quite high (thermal) inertia. Several deployed design principles optimize this issue: The TEC controller, whose electronics reacts quite sensitively to temperature change, is directly mounted with its heatsink to the chassis of SEALDH-II to increase its thermal mass. The more sensitive part is the DFB diode laser. The used laser chip is packaged (by NEL, Saddlebrook, NJ, USA) on a TEC element in a butterfly housing (see pictures in [8]) and has by itself a very small thermal mass. The NTC sensor used to measure the laser chip temperature is mounted on the same Peltier element as the laser chip, but a few millimeters away from it. Both issues lead to a quite sensitive, but temporally different response to (external) temperature changes. SEALDH-II solves that problem by adding a heatsink of approximately 200 g copper to the laser housing as an independent heatsink to increase the thermal mass. This copper heatsink with mounted laser housing is located in a small aluminum box which is primarily needed for parasitic absorption minimization (description in [8]) but also serves as a thermal shield. This copper heatsink has a weak thermal conductivity to the aluminum box and is therefore semi-insulated from the outside—good enough to convey the heat losses of the laser, weak enough to minimize coupling to fast ambient temperature changes. Figure 5 (Right) visualizes the red copper heatsink and the local, undisturbed environment (blue) in the aluminum housing (dashed black line). The inside temperature of the aluminum housing (T_air_) and its structural temperature (T_structure_) are constantly monitored to check the effectiveness of this preventive measure as well as to find possible correlations revealing further unwanted dependencies. With this approach, we achieve a final laser temperature stability level in the 10^−5^ K range, which is measured via the stability of the laser line position.

The last module in Figure 5 (Right) is the “self-learning adjustment”. This module comprises two submodules which are automatically activated and adjusted: *position average mechanism* and *add-wing information*. The first one uses the retrieved 10 Hz position information to calculate the ideal average of position information to achieve a low precision, which is done by a simplified version of the Allan variance [41] approach. The typical response time of this feedback loop is on the order of 1–2 s, in other words 10–20 positions are typically averaged. Due to the above described thermal mass concept, this module usually behaves like a sliding window algorithm with a fixed window size, except for situations where large temperature changes or other disturbances such as electromagnetic, force the module to decrease the window size. The *add-wing information module* uses the information from the wing of the low pressure absorption line profile (=transition area between absorption maximum and baseline) where the strong gradient allows an independent assessment of relative instabilities of the laser temperature and therefore of the spectral position. SEALDH-II’s final resolution of the entire locking module, however, is currently limited by the D/A converter card (16-bit) and its update frequency (140 Hz) in combination with special filter electronic acting as a virtual bit enhancer, similar to pulse width modulation (PWM). Therefore, during standard operation the add-wing information module effectively provides only monitoring information since the above described locking algorithm is precise enough to generate the feedback signals for the subsequent hardware.

### 3.3. Performance Validation

**Figure 6:**
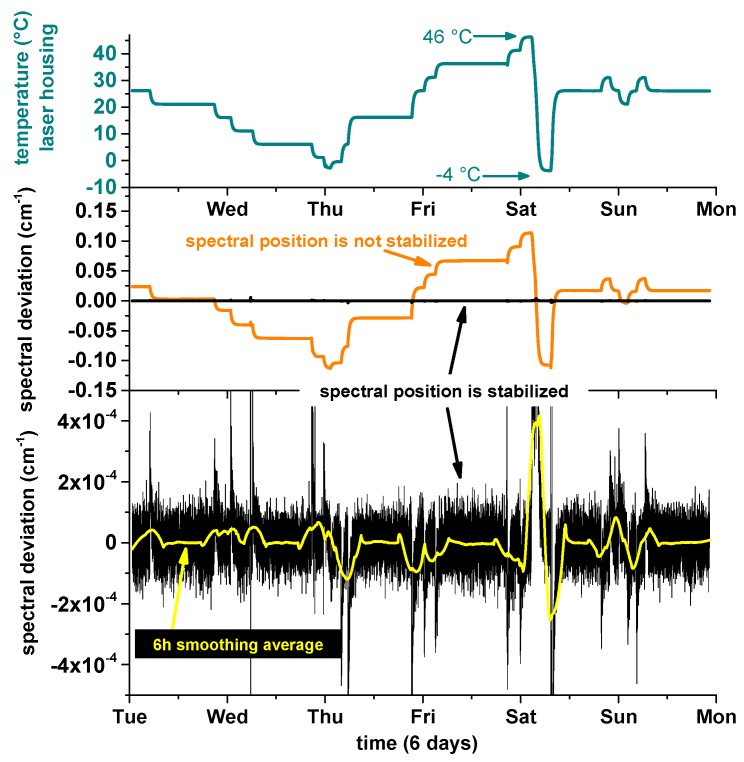
Performance test of spectral stabilization algorithm. The temperature of the spectrometer (top, blue) was variated in an environmental simulation chamber during six days. The black data in the middle subplot show the spectrally stabilized line positions, the orange data show what would have happened if no stabilization had been deployed. The bottom subplot shows a zoomed view on the black data of the middle subplot.

To test SEALDH-II’s instrument response, and as such the spectral stabilization, with respect to external temperature variations, and to derive a coefficient for the thermal stability, SEALDH-II was installed in an environmental testing chamber, which facilitated a controlled variation of the ambient temperature around SEALDH-II from −10 °C to 40 °C. The results of this five day test cycle are shown in Figure 6: here the top graph shows the temperature within the above mentioned metal housing (T_air_), which is approximately 6 K warmer due to heating elements inside of the opto-electronic laser box (described above) used for avoiding internal condensation in the gas pipes and measurement cell. Compared to the validation time scale, the temperature changes of the environmental chamber happened almost instantaneously (minutes vs. days). The T_air_ temperature data demonstrate the desired inertia effect of the above discussed thermal mass concept which conveys external temperature changes quite slowly. The black line in the middle subgraph shows the deviation between the actual spectral position in the wavelength scan and the target position, directly calculated in units of wavenumbers (cm^−1^). The lower subgraph shows the magnified view on these spectral deviations. This graph depicts 21,500 out of approx. 1.1 million retrieved data points (spectral positions)—in other words only every 50th retrieved position is shown. Clearly visible is the peak-to-peak position noise of ±1 × 10^−4^ cm^−1^ (1 Hz position data). A six hour moving average (yellow) shows that temperature changes of 5 K lead to transition deviations in the range of approx. 5 × 10^−5^ cm^−1^ until the system is in thermal equilibrium again.

**Figure 7:**
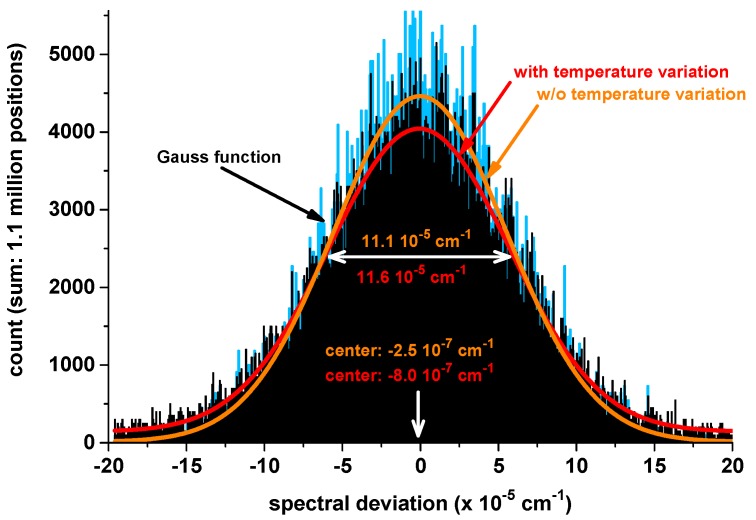
The black histogram shows the deviations between the actual and the set line position for the entire six day of validation. The blue histogram includes only the parts where the temperature of chamber and instrument were stable. The total number of line positions is scaled for comparison to the number of positions in the black histogram. The red Gaussian-shaped function corresponds to the black, the orange one to the blue histogram.

Figure 7 shows two histograms generated with the Figure 6 data. Here, the blue data in the background show the entire dataset with a fitted Gaussian profile (orange). If we remove all data from thermal transition regions where temperature changes occurred, in order to avoid dynamic effects, we generate the black data set with a red fitted Gaussian profile. To allow a better comparison of these two data sets, the black data are scaled to the same total amount of data as the blue data. The orange data in the middle subgraph (Figure 6) show what would have happened if the spectral stabilization had been deactivated. These data are retrieved from the spectral stabilization feedback (=set temperature values (T_set_ in Figure 2, Right)) in combination with the temperature tuning coefficient of the laser. This demonstrates a dampening of the ambient temperature changes impact by a factor of 340 (0.2/6 × 10^−4^) in the transition regions and by a factor of up to 800,000 (0.1/1.2 × 10^−6^) in the constant regions (divisor = uncertainty calculated above). The first number is the important one, since SEALDH-II’s underlying design target is field and aircraft operation where ambient temperature changes occur regularly; e.g., temperature change during the day, or cabin temperature change of research aircraft caused by the scientific payload and insufficient air conditioning on low flight levels.

## 4. Precision

The long-term stability test under variable environmental conditions in the next section requires a careful assessment of SEADH-II’s precision in order to distinguish real and artificial fluctuations. This is done with two approaches.

### 4.1. Optical Precision or Single Absorption Profile Analysis

A first approach can be done with the absorption profiles in Figure 3: the SNR between maximum peak absorptions (ODe_peak_) in the optical density (ODe = −ln(*I*/*I*_0_) ) and the *local* noise (σ_local_) in the residual is 400 for the 600 ppmv H_2_O concentration, and 3950 for the 8000 ppmv, respectively. In both cases, the statistical *local* noise is defined as the 1 sigma variance without the obvious systematic line shape deviation. This definition of the local noise implies that only the small variations affect the precision while the line shape deviation as a systematic effect does not. The absolute noise values at both concentrations are similar, leading to a precision of 2 ppmv at 8000 ppmv and to 1.5 ppmv at 600 ppmv, both at a temporal resolution of 7 Hz. This leads to a bandwidth and path length normalized precision of 1.1 ppmv·m·Hz^−½^ and 0.85 ppmv·m·Hz^−½^. However, it should be kept in mind that the extractive closed-path absorption cell (an improved version of the one published in [36]) has a very rigid and stiff mechanical design which leads to a quite stable fringe (=optical interference) structure. This structure can be observed in the blue residuals in Figure 3 (Left); they clearly dominate “the” noise. These fringe structures are hidden but included in the noise (σ_local_), which increases the calculated precision values, but doesn’t increase SEALDH-II’s “real” precision. Hence, these calculated single scan performance numbers should only be seen as an upper threshold.

### 4.2. System Precision via Allan Variance Analysis

**Figure 8:**
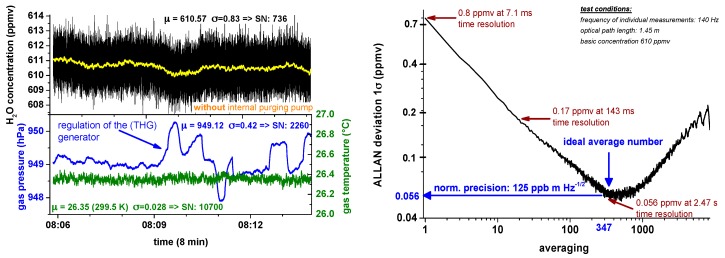
**Left**: H_2_O concentration measurements for the Allan variance plot (**Right**) with corresponding pressure and temperature values; **Right**: The Allan variance plot demonstrated under these test conditions an achieved precision of 0.8 ppmv at 140 Hz (no average), 0.17 ppmv at 7 Hz (standard operation mode of SEALDH-II) and a lowest achievable precision (=optimal precision) of 0.056 ppmv at 0.4 Hz.

A different way to assess the precision of an analytical system, here a TDLAS instrument, is the Allan [41,42] variance analysis also known as “two-sample variance”, which was initially developed to characterize the frequency stability in clocks, oscillators and amplifiers. Recently it has been more and more frequently used to characterize the system stability of TDLAS analyzers. To apply it, a stable gas concentration has to be supplied to and measured by a TDLAS spectrometer over a sufficiently long time scale of at least a few minutes with a high enough time resolution (here 140 Hz, no scan averaging). Due to this requirement, it is obvious that an “inflight Allan variance” with ambient outside air is difficult to realize. The Allan analysis should not be realized with static—i.e., zero flow—conditions but with the intended flow during a typical field experiments in order to include any flow-initiated noise effects, gas oscillations, temperature inhomogeneity, etc. Figure 8 in the left top subplot shows an Allan analysis of SEALDH-II for a stable concentration of 600 ppmv generated by a two pressure humidity generator (THG). The lower left plot shows gas pressure and temperature stability during the run of approx. 10 min: The temperature is very stable due to the previously mentioned heat exchanger and thermal mass concept implemented in SEALDH-II (max temperature drift lower than 0.1 K, 1σ 0.028 K, SNR = 10,700). The gas pressure profile (total: 1σ 0.42 hPa, SNR = 2260; local: 1σ 0.08, SNR 12,000) is mainly influenced by the pressure regulation cycles of the two pressure generator which directly affects the provided gas flow and therefore leads to slight pressure changes in SEALDH-II’s measurement cell. Both the temperature and the pressure readings have a 10 Hz time resolution.

Figure 8 (Right) shows the Allan deviation plot. In the typical 7 Hz operation mode SEALDH-II has a precision of 0.19 ppmv H_2_O (0.17 ppmv·m·Hz^−½^). The optimum precision of 0.056 ppmv (0.125 ppmv·m·Hz^−½^) is reached at 0.4 Hz temporal resolution. These results justify the primary contention about the stability of the *local* noise (σ_local_) in Figure 3. Based on these results, we can deduce a purely statistical noise contribution with a magnitude of about 2 × 10^−5^ in the *local* noise from Figure 3. This also means that most of the *local* noise (i.e., 2 × 10^−4^) in Figure 3 is caused by more or less static, systematic baseline variations like etalon fringes, which are caused by the miniature multi-path White cell of SEALDH-II and which cannot be reduced by scan averaging. As a remark, it should be mentioned, that these results may also be an upper threshold since the H_2_O fluctuations in the gas stream caused by the generator could increase the ideal precision value. Therefore, the true precision value of SEALDH-II is probably even better. 

## 5. Effects of Ambient Temperature and Humidity Variations

As described in the previous chapter, SEALDH-II is equipped with a broad range of internal measures to prevent, minimize, or at least monitor signal influences in those scenarios which might occur in-flight. Ambient temperature and humidity variations are in typical deployments the most important and most common external disturbances in field situations which influence the dTDLAS response. Direct short-term temperature influences are minimized by the thermal mass concept in SEALDH-II, but this still has to be validated. This holds even more for long-term variations. Hence, the SEALDH-II validation run had to be set up in a way that SEALDH-II was actually exposed to both long-term and short-term changes. We decided to measure one H_2_O concentration value (500 ppmv), at one pressure (925 hPa) for 6 days under various environmental conditions. A critical part for the validation was to guarantee that the external reference value was defined with a low uncertainty. Here we used a traceable humidity generator (THG) with a metrological uncertainty of 0.42% (=2.1 ppmv at 500 ppmv). As a comparison, the above mentioned international comparison campaign AquaVIT [5] didn’t have a metrological reference value at all, which yielded the problem that the large deviations (±10%) between the instruments could not be explained.

### 5.1. Traceable Humidity Generator (THG)

**Figure 9:**
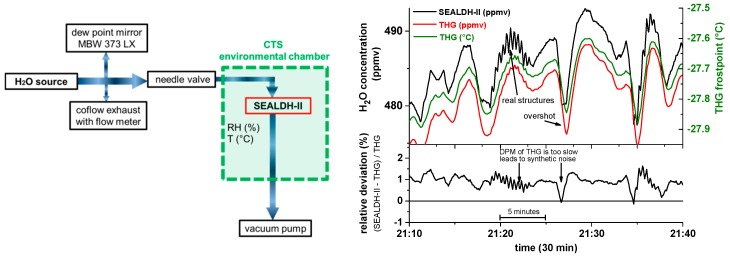
**Left**: Setup for validation of SEALDH-II’s response to a variation in ambient temperature and humidity; **Right:** Detailed view on the results. The traceable humidity generator (THG) has a highly defined absolute accuracy but within the uncertainty an undefined short-term behavior. The precision of SEALDH-II is high enough to resolve these structures, while the THG’s internal dew point mirror hygrometer (DPM) is too slow to resolve them. This yields to synthetic noise in the relative deviation between SEALDH-II and THG.

**Figure 10:**
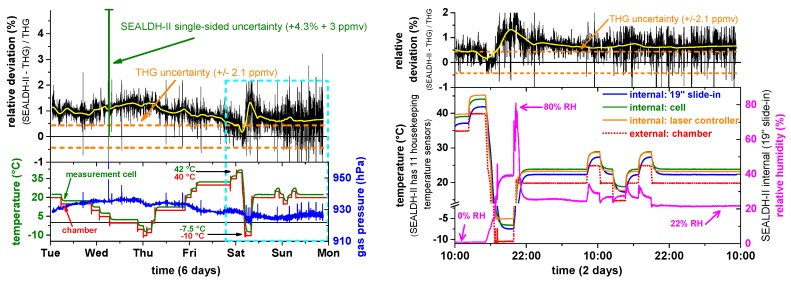
Validation results from the ambient temperature and humidity variation. **Left:** The black data show the relative deviation between the traceable humidity generator (THG) and SEALDH-II with SEALDH-II’s (green) and the THG’s (orange) uncertainty; **Right:** The bottom subplot shows the ambient (= internal chamber) temperature (red), gas temperature (green) and gas pressure (blue) in SEALDH-II’s measurement cell. The left subplot shows the zoomed section where also the ambient humidity (magenta) is varied.

The THG employs the well-known two pressure generator principle [43], here with an ice saturation unit, described in [44]. The traceable link between the wetted air flow and the generator is established by adding (Figure 9, (Left)) a primary calibrated dew point mirror hygrometer (DPMH) [45] as a humidity monitor. The uncertainty of the DPMH at this 500 ppmv calibration point is ±2.1 ppmv or 0.42%. The “coflow”-exhaust outlet (Figure 10, (Left)) allows operating the humidity generator and the DPMH at their ideal flow rates (5 and 3 SLPM respectively) to achieve lowest uncertainties, which are close to the uncertainty of the German national primary humidity standard (approx. 0.3%). Figure 9 (Right) shows a detailed measurement section of 30 min: The top graph depicts the short-term behavior of the THG (with fluctuations on the order of ±1% caused by generation adjustment cycles). The green line shows the frost point measurement of the DPMH, which is used to calculate the final H_2_O concentration reference value (red). In black, the SEALDH-II measurement is shown.

The DPMH typically has a relatively slow (temperature dependent) response time in the minute range which prevents detection of fast H_2_O variations such as around 21:20. These fast H_2_O fluctuations are real, because they can be seen in both the smoothed residual spikes on the THG values and in the SEALDH-II measurements. However, SEALDH-II has a precision of 0.056 ppmv and a fast response which allows detecting these fluctuations with a SNR of about 100. The relative deviations between SEALDH-II and THG (see lower right panel in Figure 9 (Right)) reveal that these different temporal resolving capabilities lead to “synthetic noise”, which will also be visible in the following graphs (Figure 10).

### 5.2. External Influence Validation

For validating SEALDH-II’s stabilization mechanisms and assessing the most important external influence effects, SEALDH-II was installed inside an environmental climate chamber large enough to uptake the entire spectrometer (see Figure 9). The chamber allowed varying the relative humidity from 0%–80% rH and the gas temperature from −10 to 40 °C. The THG provided a net gas flow of approx. 3.5 L/min to SEALDH-II.

### 5.3. Validation Results

Data from the validation experiment are shown in the left bottom graph of Figure 10. Here, the chamber temperature (red trace) was varied stepwise between −10 °C and 40 °C. SEALDH-II’s temperature in the measurement cell (green trace) is approximately 2 K higher as a result of the active heating inside the SEALDH-II instrument installed to prevent condensation within the gas ducts or the optical cell under all circumstances. The blue trace shows the gas pressure inside the measurement cell which is quite constant around 925 hPa. The upper graph in Figure 10 (Left) shows the relative deviation between the humidity values derived with SEALDH-II and those from the THG. The calculated uncertainty of SEALDH-II (4.3% ± 3 ppmv) is marked with the green half-side uncertainty bar, the THG uncertainty of ±2.1 ppmv is marked in orange. Despite the noise, one can see a systematic structure in the relative deviation which correlates to a large degree with the temperature variations of the chamber. The average relative deviation is 0.9%, with a largest deviation of 1.4% and a smallest of 0.1%. The largest deviation difference of 1.3% occurred when the largest temperature change was initiated from 40 °C to −10°C (Saturday). From this structure, we can estimate the temperature coefficient of SEALDH-II with respect to external temperature changes to be as small as 0.026%/K, which indicates the excellent effectiveness of the stabilization algorithm. Nevertheless, it is also important to note that the temperature induced deviations remain much smaller (a factor of four) than SEALDH-II’s uncertainty. This was to be anticipated, as SEALDH-II’s overall uncertainty was calculated conservatively, based on the physical model (Equation (2)). On the other hand, it also has to be noted that SEALDH-II is so stable that even quite significant temperature variations do not push the transfer function outside its uncertainty limits.

A magnified subsection (blue dotted area) of this data is displayed on the right side of Figure 10. Here the top graph shows again the relative deviation between SEALDH-II and THG, while the bottom graph shows selected, representative temperature measurements within the SEALDH-II structure. The effect of the passive thermal inertia optimization is clearly visible: While the temperature variation of the climate chamber (red) shows steep edges, all internal SEALDH-II components react quite slowly and are strongly damped with more than an hour time delay with respect to the external temperature change. The laser driver electronics (orange) shows one of the highest temperatures, due to its internal heat dissipation, followed by the actively heated optic module (green) including the closed-path absorption cell and by the internal slide-in (blue). These data demonstrate that SEALDH-II is highly immune against fast as well as slow temperature changes. The temperature coefficient level of 0.026%/K is therefore valid for all situations, including the deployment in an aircraft cabin.

Figure 10 also shows that SEALDH-II is entirely immune against external relative humidity (RH) changes. In the first 4.5 days, the air was dried (RH close to 0%), in the last two days, the RH value was varied. Overall, no systematic correlation could be deduced (coefficient < 0.001% per %RH change). This excellent result is a consequence of the novel and extensive parasitic water vapor compensation methods included in SEALDH-II which are described in detail elsewhere [8].

## 6. Conclusions

The new SEALDH-II diode laser hygrometer described in this paper introduces a novel class of entirely controlled/stabilized diode laser absorption spectrometers. The core of this instrument is a first-principles extractive, closed-path, 1.4 μm TDLAS hygrometer which does not need to be calibrated within its uncertainty range. The instrument covers a concentration range from 3 to 40,000 ppmv with an optimum precision of 0.056 ppmv at 0.4 Hz (i.e., 0.125 ppmv·m·Hz^−½^) and a calculated uncertainty of 4.3% ± 3 ppmv. Its data evaluation is based on first principles and avoids any typical calibration related issues, which are currently seen as one of the most important contributions to the persistent deviations between airborne hygrometers. SEALDH-II comprises several novel sub-functions to ensure that validation results from a laboratory can be transferred to harsh environments such as field deployment or even aircraft. This includes monitoring, correcting functions, and deployment of preventive measures to minimize impacts from ambient environment changes (i.e., temperature, pressure or relative humidity), aging, wear and tear, internal influences, or user interactions. One of the important internal control mechanisms is a robust and intelligent spectral stabilization, which was described in detail and also validated under large and fast ambient temperature variations (Δ = 50 K). This module improved the accuracy of the absolute spectral tuning range, compared to a non-stabilized instrument, by a factor of 340. Furthermore, we demonstrated a long-term spectral locking accuracy in the 10^−4^ cm^−1^ range, which allows using a pre-calculated line position in the main absorption line fitting module. This results in a reduction of free fitting parameters, which significantly increases the stability of the fit-based evaluation process. Finally, SEALDH-II (and its operating modules) was comprehensively validated in an environmental chamber. Despite the large relative humidity (0%–80% RH) and temperature (−10–40 °C) variations, the instrument remained under all circumstances within its stated uncertainty range and showed only tiny temperature triggered output variations. The total temperature coefficient is determined to only 0.026%/K and no humidity dependence (with an upper threshold coefficient of 0.001%/%RH) could be measured. Therefore, SEALDH-II is capable of handling the typical external disturbance ranges with negligible deviations, which clearly indicates its suitability for harsh environments, which have frequently led to unexplainable, inconsistent datasets between instruments.

SEALDH-II is therefore well suited for field campaign use on the ground as well as in airborne applications and to serve as a metrological reference for field validation. To further investigate these options, it is planned that SEALDH-II will participate in a long-term (12 months and more) behavior study to characterize and improve datasets sustainably.

## Figures and Tables

**Table 1:** This Table describes different scenarios which could happen during operation, e.g., an ambient humidity change (first division, third line) can affects accuracy “a” and precision “p”, lead to a system crash “c” or even cause permanent damages “d” to the instrument. SEALDH-II monitors “m” this value, corrects the final measurement values for this effect “cor” and has preventive measures deployed “p” to keep the corrections a small as possible. A “(cor)” in the table means that corrections can be done in many cases, but not in all, e.g., related to software crashes this means that several software exceptions are handled but, similar to every computer system, not every scenario can be handled.

Scenario	Accuracy/Precision & Damage/Crash	Monitored & Corrected & Preventive Measures
**Ambient Impact**		
temperature	a p & d c	m & cor & p
pressure	a p	m
humidity	a p & d c	m & cor & p
electromagnetic radiation or emission	a p & d c	m & p
power supply issues	a p & d c	m & p
vibrations/mechanical stress	a p & d c	m & p
**Aging/Wear and Tear**		
change of laser charateristics	a p	m
changes in optical setup	a p	m & p
variation of fringe structure	a	m
sensor drift	a & d	m & (cor) & p
electronic drift	a & d	m & (cor) & p
electromechanical parts	a & d c	m & p
**Internal Influences**		
gas temperature inhomogenties	a p	m & p
software malfunctions		m & (cor) & p
electromechanical malfunctions		m & p
recognition of critical system condition	d c	m & p
**User**		
wrong usage of device		m & p
problems with interfaces		m & p
use without knowledge of the details	a p & d	p
online access—view on system parameters		p
